# The effect of fresh IVF cycle characteristics on frozen embryo transfer (FET) outcomes

**DOI:** 10.5935/1518-0557.20190074

**Published:** 2020

**Authors:** Nayla J. Bushaqer, Noor N. Alkhudhairy, Ziyad M. Alturaigi, Rowaida M. Alhamad, Wadha A. Mohawesh, Fatema E. Alraka, Hisham A. Ayyoub, M. Dayou Nawal

**Affiliations:** 1 Saudi Board of OB/GYN; 2 Infertility and IVF Saudi fellowship; 3 Bahrain Defense Force (BDF) Hospital, Riffa, Bahrain; 4 IVF unit, Prince Sultan Military Medical City (PSMMC), Riyadh, KSA; 5 King Fahad Military Medical Complex (KFMMC), Dammam, KSA; 6 Prince Sultan Military Medical City (PSMMC), Riyadh, KSA; 7 Prince Sultan Military Medical City, Riyadh, KSA; 8 The Royal College of Surgeons in Irland MUB, Bahrain

**Keywords:** frozen embryo transfer (FET), cryopreservation, thawing, in vitro fertilization (IVF), Intra-Cytoplasmic Sperm Injection (ICSI)

## Abstract

**Objective:**

To determine the effect of fresh IVF/ICSI cycles on FET cycle embryo and pregnancy outcomes.

**Methods:**

This retrospective cohort study included data from the medical records of 104 FET cycles performed from January 2014 to December 2016. Embryos were previously vitrified and then thawed for embryo transfer. Statistical significance was established at *p*<0.05. The main endpoints were FET cycle survival and pregnancy rates.

**Results:**

A total of 104 FET cycles were analyzed for survival; 94 showed good progression and 84 achieved embryo transfers. Patients with secondary infertility achieved significantly higher pregnancy rates - 6/38 (15.8%) *vs.* 18/46 (39.1%) (*p*<0.018). Stimulation with FSH/LH resulted in more significant embryo progression, 38/48 (79.2%) *vs.* 28/46 (60.9%) in the FSH group (*p*=0.01). Patients who got pregnant from fresh cycles had the highest pregnancy rates in FET cycles (*p*<0.0001). Lower numbers of frozen embryos correlated with higher pregnancy rates (*p*=0.048). Embryos frozen on day 2 or 3 had the most significant progression (*p*<0.0001). Freeze-thaw intervals >12 months yielded higher pregnancy rates, 13/30 (43%), *vs.* 11/54 (20.4%) (*p*=0.025).

**Conclusion:**

Patient pregnancy in fresh cycles is a good prognostic factor for FET cycle success. Delaying FET by more than 12 months might result in higher pregnancy rates.

## INTRODUCTION

Cryopreservation enables non-implanted embryos generated from in-vitro fertilization/intra-cytoplasmic sperm injection (IVF/ICSI) cycles to be stored and used at a later time (Groenewoud *et al*., 2013; 2017). They account for about 20% of all embryo transfers (ETs) in Europe and throughout the world (Han *et al*., 2012; Zheng *et al*., 2014). Cryopreservation was introduced in 1983 and has since become very popular and important in assisted reproductive technology (ART) (Han *et al*., 2012; Zheng *et al*., 2014; Burks *et al*., 2015; Wong *et al*., 2014; Santos-Ribeiro *et al*., 2016; Eftekhar *et al*., 2013; Boostanfar *et al*., 2016; Davar *et al*., 2016; Bdolah *et al*., 2015). Embryo cryopreservation technology has undergone significant improvements since its introduction, leading to better outcomes from frozen embryo transfers (FET). The progress seen in embryo cryopreservation has encouraged its routine use in ART centers (Ozgur *et al*., 2016).

Cryopreservation of embryos may be performed at different embryo development stages, including the pronuclear, cleavage, or early-expanded blastocyst stages (Bdolah *et al*., 2015). Slow freezing and vitrification are the preferred methods (Bdolah *et al*., 2015; Basirat *et al*., 2016). The popularity of vitrification has increased in IVF centers due to its ease of implementation, reduced procedure time, and success rates (Han *et al*., 2012; Van Landuyt *et al*., 2013; Lopes *et al*., 2015; Guo *et al*., 2013; Cercas *et al*., 2012). It prevents ice formation and decreases the rate of cooling damage to the cells (Basirat *et al*., 2016; Li *et al*., 2014). Slow freezing also requires expensive equipment and much more time -approximately one to two hours compared to several minutes in vitrification (Han *et al*., 2012; Basirat *et al*., 2016).

Cryopreservation has significantly expanded the possibilities of ART. It allows women producing a large number of oocytes to store embryos, thus decreasing multiple pregnancy and embryo wastage rates (Zheng *et al*., 2014; Wong *et al*., 2014; Ozgur *et al*., 2016; Basirat *et al*., 2016; Guo *et al*., 2013; Ashrafi *et al*., 2011; Peeraer *et al*., 2015; Imbar *et al*., 2012). It has also been used to prevent ovarian hyperstimulation syndrome (OHSS) and defer ET in high risk patients (Zheng *et al*., 2014; Wong *et al*., 2014; Boostanfar *et al*., 2016; Ozgur *et al*., 2016; Basirat *et al*., 2016; Ashrafi *et al*., 2011; Peeraer *et al*., 2015; Basile & Garcia-Velasco, 2016; Blockeel *et al*., 2016; Kassab *et al*., 2009; Zhu *et al*., 2015). Cryopreservation plays a pivotal role in poor candidates for fresh embryo transfers due to inadequate endometrial preparation or receptivity (Santos-Ribeiro *et al*., 2016; Eftekhar *et al*., 2013; Guo *et al*., 2013; Peeraer *et al*., 2015; Blockeel *et al*., 2016; Roque *et al*., 2015). The procedure has also been implemented to increase the use of preimplantation genetic diagnosis (PGD) and store non-implanted tested and desired embryos (Basile & Garcia-Velasco, 2016; Blockeel *et al*., 2016). Embryo cryopreservation offers patients diagnosed with cancer a chance to store and use their frozen embryos in the future (Wong *et al*., 2014; Kassab *et al*., 2009; Barcroft *et al*., 2013).

FET cycles have yielded lower pregnancy and implantation rates in comparison to fresh IVF/ICSI cycles (Guo *et al*., 2013; Ashrafi *et al*., 2011; Peeraer *et al*., 2015) for two main reasons. First, fresh ET relies on the selection of the best embryos, while lower quality embryos are saved for freezing. Second, ice crystals formed during freezing and thawing may cause adverse effects on embryos (Ashrafi *et al*., 2011; Peeraer *et al*., 2015; Eftekhar *et al*., 2014). Nonetheless, some authors have reported similar or higher pregnancy rates from FET compared with fresh transfer cycles (Zhu *et al*., 2015; Veleva *et al*., 2013).

Pregnancy outcomes following FET are thought to depend on several clinical factors, including the age of the woman at the time of embryo cryopreservation (Davar *et al*., 2016; Bdolah *et al*., 2015; Kassab *et al*., 2009; Eftekhar *et al*., 2014; Veleva *et al*., 2013); the duration, cause, and type of infertility (primary or secondary); endometrial thickness on the day of embryo transfer (Davar *et al*., 2016; Eftekhar *et al*., 2014); the endometrial preparation protocol; success of a previous fresh cycle (Kassab *et al*., 2009); follicle stimulating hormone (FSH) levels; and the reason for embryo cryopreservation (Eftekhar *et al*., 2014). Additional technical and embryological factors include the oocyte fertilization method (IVF/ICSI) used (Davar *et al*., 2016; Eftekhar *et al*., 2014); the freeze-thaw interval (Kassab *et al*., 2009); the phase of embryo development at freezing; the degree of embryo damage after thawing; and embryo progression after thawing (Davar *et al*., 2016).

This paper aimed to describe the effects of the clinical variables tied to first IVF cycles/fresh ET on the outcomes of FET cycles.

## MATERIALS AND METHODS

### Study design and setting

The charts of all patients submitted to frozen embryo transfers (FET) from January 2014 to December 2016 were manually reviewed after the Prince Sultan Military Medical City (PSMMC) Research Ethics Board approved the study design. A total of 104 cycles were included in our retrospective study. The study was performed in accordance with the principles of the Helsinki Declaration.

### Inclusion and exclusion criteria

All patients submitted to FET cycles were included, regardless of age. The individuals offered fresh cycles were aged 35 years or younger. The patients offered FET had the procedure regardless of their age at the time of embryo thawing. Patients without adequate endometrial thickness for FET were excluded; their embryos were not thawed or transferred. Patients who did not have frozen embryo transfer were also excluded.

### Controlled ovarian stimulation

All patients had previously had fresh cycles (IVF/ICSI) regardless of the freeze-thaw interval. Controlled ovarian stimulation (COS) in fresh cycles was performed with the aid of rFSH (Gonal-f, Merck, NJ, USA) or human menopausal gonadotropin (HMG) (Menogon, Ferring, Saint-Prex, Switzerland). In the long protocol, a gonadotropin- releasing hormone (GnRH) agonist was started on day 21 of the previous menstrual cycle (Decapeptyl 0.1 mg/day, IPSEN, Paris, France); in the short protocol, Decapeptyl 0.05mg/ day was started on day 1 of the stimulation. Another alternative was the fixed GnRH antagonist protocol (Cetrotide 0.25 mg/day, Merck, NJ, USA), started on day 6 of stimulation. The cycles were monitored through serial vaginal ultrasound scans and serum levels of estradiol (E2) and FSH. Whenever needed, the rFSH/hMG dosages were adjusted based on ovarian response. When two or more dominant follicles reached a mean diameter ≥ 18 mm or three or more reached a mean diameter of ≥ 17 mm, the patients were administered 5-10,000 IU hCG. The oocytes were retrieved 36 hours after hCG injection (Pregnyl, Merck, NJ, USA).

### Embryo quality assessment

The embryos were categorized based on cleavage stage, fragmentation, blastomere size, shape, and number. They were frozen for different reasons, including excess embryos, prevention of ovarian hyperstimulation syndrome (OHSS), or because the patient had too thin endometrium for FET. The embryos were cryopreserved with the consent of the couples for one year, subject to extension for another year when they were not used. Embryos were discarded if a member of the couple died, if the couple got a divorce, or if they requested that their embryos were discarded.

Cleavage stage embryos and blastocysts were cryopreserved on an open vitrification system using MediCult Vitrification media and the Cryoleaf method (Origio, Denmark) on days 2 to 5 after retrieval. The process was performed at room temperature, and the steps were carried out according to manufacturer instructions. No less than two embryos were cryopreserved, and usually two to four embryos were loaded onto one Cryoleaf.

### Endometrial preparation

During the FET cycle, patients were randomly assigned to one of two endometrial preparation protocols, namely hormonal replacement therapy (HRT) or minimal stimulation protocol (MSP) starting on days 2-5 of the menstrual cycle. Patients were offered a preparation protocol if endometrial thickness was ≤5 mm.

In the HRT protocol, 2-mg oral estradiol was given twice daily with Decapeptyl (0.05 mg/day) for 6 to 7 days. The patients subsequently underwent ultrasound examination for endometrial thickness and ovarian follicle formation. When endometrial thickness reached ≥7 mm, Cyclogest 400 mg vaginal progesterone pessaries (L.D.COLLINS & CO., London, UK) were given to the patients twice daily.

The patients offered MSP were administered intramuscular injections of Menogon 75 IU for six to seven days. Then they underwent ultrasound examination to assess endometrial thickness and ovarian follicle formation. When endometrial thickness reached ≥7 mm, decapeptyl was discontinued and Cyclogest 400 mg vaginal progesterone pessaries (L.D.COLLINS & CO., London, UK) were given to the patients twice daily.

### Embryo thawing

The Medicult Vitrification Warming method (Origio, Denmark) was used in embryo thawing. Embryos at cleavage stage were thawed the day before FET and transferred the following day. Blastocysts were thawed in the morning of FET and transferred on the same day. The usual practice is to thaw one embryo straw, and at least half of the blastomeres should be intact on thaw day and accounted for in terms of survival rate. If survival is <50%, another embryo straw is thawed when available.

Embryo progression was defined as division of embryos from day 2 and day 3 on the following day. If the embryos failed to show signs of progression on the day after thawing, another straw was thawed - if available - and the best embryos were transferred on the same day to maximize the chances of success.

### Embryo transfer

Embryo transfer took place mostly on the fourth day of progesterone administration in an attempt to synchronize embryo age and the day of transfer, considering the day of progesterone initiation as day 0 of embryo age. A maximum of three embryos were transferred under ultrasound guidance. Patients were kept on Cyclogest pessaries until they were tested for pregnancy or for up to 12 weeks if they became pregnant. Pregnancy tests were considered positive when serum hCG was ≥10 ml IU/ml 12 days after embryo transfer. Transvaginal ultrasound examination was performed two weeks after a positive pregnancy test to confirm the existence of intrauterine pregnancy, identify the number of gestational sacs, and verify fetal viability.

### Data collection

Fresh cycle data included patient age at the time of the fresh cycle, BMI, parity and previous miscarriages, type and cause of infertility, previous IVF or ICSI, COS protocol, drug used in stimulation, and endometrial thickness with quality grading (grade b being the best, and grade a the worst). The following grading scheme was used in the assessment of endometrium quality: grade a was assigned to homogeneous, hyperechoic endometria; grade b was assigned to endometria showing a triple line pattern made up of two hypoechoic layers and a central hyperechoic layer; grade c was assigned to endometria with an intermediate iso-echogenic pattern. Pregnancy tests were recorded from the fresh cycles.

FET data included total number of frozen embryos, day of freezing, indication of freezing, and freeze-thaw interval. The total number of embryos thawed and transferred, day of FET, and thawed embryo survival rate and progression were also recorded.

Databases PubMed and MEDLINE were searched for papers published previously on the topic.

### Statistical analysis

The StatsDirect statistical package (version: 3.0.141 Cheshire UK 2015) was used in data analysis. Variance was compared by means of the two-tailed F test. Since no significant difference was found in the F test, the variables were considered to follow a normal distribution. An unpaired two-tailed T test was used to assess the differences in thawed embryo mean survival between two groups. One-way ANOVA was used to assess the difference in mean survival between more than two groups. The Chi-square test was used to assess the proportion of cleaved embryos after survival and pregnancy rates. The Fisher-Freeman-Halton exact test was used in crosstabs when a cell had an expectation of less than 5. *p*-values of less than 0.05 were considered statistically significant.

## RESULTS

Our study included 104 FET cycles with a pregnancy rate of 34%. The thawed embryos from 94 of the 104 FET cycles survived, and 84 cycles were performed with cleaved embryos and led to embryo transfers. The pregnancy rate for initiated FET cycles was 23% (24/104). The pregnancy rate for embryo transfers was 29% (24/84).

A series of variables pertaining to fresh cycles were analyzed for possible connections with embryo survival, progression, and pregnancy rates. No effect was found for patient age, BMI, cause of infertility, and previous live birth or miscarriage. Patients with secondary infertility had significantly higher pregnancy rates compared with the patients with primary infertility [18/46 (39.1%) *vs.* 6/38 (15.8%); *p*<0.018] (Table 1).

**Table 1:** The effect of fresh cycle patient characteristics on thaw cycle outcomes

	Survival rate Mean ± SD Total cases n=104	*p*-value	Progression rate N (%) TotalSurvived n=94	*p*-value	Pregnancy rate N (%) Transferred n=84	*p*-value
Patient age:		0.9*		0.89***		0.49****
<35 years	83.7±34.7		56/81 (69.1%)		22/72 (30.6%)	
≥ 35 years	82.5±35.9		10/13 (76.9%)		2/12 (16.7%)	
Patient BMI:		0.07*		0.27***		0.83***
<30 kg/m^2^	79.3±38		40/62 (64.5%)		15/54 (27.8%)	
≥30 kg/m^2^	92.2±25		26/32 (81.3%)		9/30 (30%)	
Type of infertility:		0.88*		0.74***		0.018***
Primary	82.9±35.8		29/44 (65.9%)		6/38 (15.8%)	
Secondary	84±34		37/50 (74%)		18/46 (39.1%)	
Cause of infertility:		0.15*		0.06***		0.15***
PCO	79.2±38.6		31/51 (60.8%)		9/42 (21.4%)	
Non PCO	88.9±28.9		35/43 (81.4%)		15/42 (35.7%)	
History of previous delivery	86.8±31.3	0.45*	28/37(75.7%)	0.73***	14/35 (40%)	0.05***
No previous delivery	81.5±36.8		38/57 (66.7%)		10/49 (20.4%)	
History of miscarriage	81.9±36.4	0.77*	15/23 (65.2%)	0.72***	8/21 (38.1%)	0.26***
No previous miscarriage	84.2±34.3		51/71 (71.8%)		16/63 (25.4%)	

* Unpaired T test

*** Chi square test

**** Fisher-Freeman-Halton exact

Furthermore, stimulation cycle parameters revealed that the type of stimulation protocol used (GnRH agonist vs. antagonist), endometrial thickness (≤1 or >1 cm), endometrial grading, and indication of freezing did not have any effect on embryo survival, progression, or pregnancy rates. Stimulation with FSH/LH (luteinizing hormone) yielded higher levels of thawed embryo progression than FSH alone [38/48 (79.2%) *vs.* 28/46 (60.9%); *p*=0.01]. Patients who became pregnant after fresh cycles had higher pregnancy rates in FET compared with patients who did not become pregnant after fresh cycles and who did not achieve ET [18/32 (56.3%), 4/33 (12.1%), and 2/19 (10.5%), respectively; *p*<0.0001] (Table 2).

**Table 2:** The effect of fresh cycle stimulation characteristics on thaw cycle outcomes

	Survival rate Mean ± SD Total cases n=104	*p*-value	Progression rate N (%) TotalSurvived n=94	*p*-value	Pregnancy rate N (%) Transferred n=84	*p*-value
Protocol type:		0.87**		0.94****		0.58****
Antagonist	84.9±33.4		17/23 (73.9%)		4/21 (19.1%)	
Long agonist	82.6±36		45/66 (68.2%)		19/59 (32.2%)	
Short agonist	90±22.4		4/5 (80%)		1/4 (25%)	
Stimulation drug:		0.05*		0.01***		0.57***
FSH	76.9±38		28/46 (60.9%)		10/39 (25.6%)	
FSH/LH	90.1±29.7		38/48 (79.2%)		14/45 (31.1%)	
Endometrial thickness		0.96*		0.62***		0.84***
≤1cm	83.4±33.8		32/44 (72.7%)		11/40 (27.5%)	
> 1cm	83.7±35.8		34/50 (68%)		13/44 (29.6%)	
Endometrial grading:		0.61**		0.98***		0.35****
A	86.6±30.6		7/10 (70%)		4/10 (40%)	
B	87.6±30.5		22/32 (68.8%)		9/26 (34.6%)	
C	80.6±37.8		37/52 (71.2%)		11/48 (22.9%)	
Indication of freezing:		0.73**		0.84****		0.05***
Excess	82.4±35.7		48/68 (70.6%)		22/62 (35.4%)	
OHSS	85.8±33.3		16/24 (66.7%)		2/20 (10%)	
Thin endometrium	100±0		2/2 (100%)		0/2 (0%)	
Outcome of 1^st^ cycle:		0.78**		0.68***		<0.0001****
No pregnancy	85.7±31.8		24/36 (66.7%)		4/33 (12.1%)	
Pregnancy	80.5±38.3		27/35 (77.1%)		18/32 (56.3%)	
No ET	85.2±33.8		15/23 (65.2%)		2/19 (10.5%)	

* Unpaired T test

** one way ANOVA

*** Chi square test

**** Fisher-Freeman-Halton exact

The laboratory data showed that the IVF technique (ICSI/IVF/split) did not have any effect on embryo survival, progression, or pregnancy rates. Patients with <5 embryos frozen had higher pregnancy rates than individuals with 5-9 or ≥10 embryos [18/46 (39.1%), 5/25 (20%), and 1/13 (7.7%), respectively, *p*=0.048]. Embryos frozen on days 2 or 3 had more significant progression [37/48 (77.1%) and 24/31 (77.4%), respectively] compared with embryos frozen on days 4 or 5 [5/7 (71.4%) and 0/8 (0%), respectively] (*p*<0.0001). Patients with freeze-thaw intervals >12 months had higher pregnancy rates than patients with freeze-thaw intervals ≤12 months [13/30 (43%) *vs.* 11/54 (20.4%); *p*=0.025] (Table 3).

**Table 3:** The effect of fresh cycle laboratory characteristics on thaw cycle outcomes

	Survival rate Mean ± SD Total cases n=104	*p*-value	Progression rate N (%) TotalSurvived n=94	*p*-value	Pregnancy rate N (%) Transferred n=84	*p*-value
IVF technique:		0.69**		0.07****		0.11****
ICSI	81.8±36		46/67 (68.7%)		19/58 (32.8%)	
IVF	89.5±31.5		15/18 (83.3%)		5/17 (29.4%)	
Split	85.3±31.9		5/9 (55.6%)		0/9 (0%)	
Number of frozen embryos		0.15**		0.97***		0.048****
<5	78±38.9		36/52 (69.2%)		18/46 (39.1%)	
5-9	91.7±26.3		20/27 (74.1%)		5/25 (20%)	
≥10	91±26.7		10/15 (66.7%)		1/13 (7.7%)	
Day of freezing:		0.13**		<0.0001****		0.74****
2^nd^	87.5±32.3		37/48 (77.1%)		15/44 (34.1%)	
3^rd^	87±29.9		24/31 (77.4%)		7/29 (24.1%)	
4^th^	64.6±44.7		5/7 (71.4%)		1/5 (20%)	
5^t^^h^	68.2±46.2		0/8 (0%)		1/6 (16.7%)	
Freezing thaw interval:		0.85*		0.83***		0.025***
≤ 12 months	83±34.8		41/58 (70.7%)		11/54 (20.4%)	
> 12 months	84.4±34.9		25/36 (69.4%)		13/30 (43.3%)	

* Unpaired T test

** one way ANOVA

*** Chi square test

**** Fisher-Freeman-Halton exact

## DISCUSSION

This study looked into the effects of fresh cycle parameters on the outcome of FET cycles, a matter not fully addressed in the literature. Usually, factors of the same FET cycle are studied, not the original fresh ET cycle characteristics.

We found that age at ovum pick up (OPU) did not affect embryo post-thaw survival, progression, or pregnancy outcomes. This may be due to the fact that fresh cycles were offered only to patients aged 35 years or younger. Other authors have described similar findings. El-Toukhy *et al.* (2003) reported that age at cryopreservation did not affect embryo survival, and Cercas *et al*. (2012) reported that it did not affect embryo progression. On the other hand, Bdolah *et al*. (2015) reported that younger patients at OPU had higher live birth rates (LBRs) from FET cycles.

Our study found that the type of infertility did not affect embryo survival or progression, and that patients with secondary infertility had higher pregnancy rates. Patients who became pregnant earlier either spontaneously or with the aid of ART were more prone to becoming pregnant, indicating that patients who never got pregnant might have an intrinsic known - male or female - or unknown factor for infertility. In regard to embryo survival, El-Toukhy *et al.* (2003) reported that the type of infertility did not affect embryo survival. By their turn, others found that being pregnant before the FET cycle did not affect pregnancy rates after an FET cycle (Bdolah *et al*., 2015; Eftekhar *et al*., 2014). Accordingly, we also found that the cause of infertility did not have any effect on FET cycle outcome. Likewise, others have reported that it did not affect embryo survival, progression (Cercas *et al*., 2012; El-Toukhy *et al.*, 2003), or pregnancy outcome (Bdolah *et al*., 2015; Ashrafi *et al*., 2011).

In terms of fresh cycle parameters and their effects on the FET cycle, we found that stimulation via GnRH antagonist or agonist protocols did not produce a significant difference on post-thaw embryos or pregnancy outcomes. Other studies have similarly reported that the fresh cycle protocol did not affect the pregnancy outcomes of FET cycles performed later (Bdolah *et al*., 2015; Eftekhar *et al*., 2012). Ashrafi found that patients stimulated with a GnRH-agonist long protocol had higher pregnancy and implantation rates than patients stimulated via a GnRH-antagonist protocol (Ashrafi *et al*., 2011). Conversely, another study found that patients stimulated with a GnRH-antagonist protocol in fresh cycles had higher live birth rates than individuals given a GnRH-agonist protocol (23.3% *vs.* 14.6%) (Toftager *et al.,* 2017).

When fresh cycles were compared for the type of gonadotropin used, we found that FSH and FSH/LH combined did not affect embryo survival or pregnancy rates, as described in a previous study (Oehninger *et al*., 2000). We observed more significant embryo progression when LH/FSH combined was administered than when FSH alone was prescribed, although this may have been an incidental finding. Nonetheless, pregnancy rates remained unchanged. Ziebe *et al*. (2007) found that embryo survival and progression improved when LH/FSH was prescribed compared with FSH alone, although live birth rates remained at 9% for both regimens in the first FET cycle after a fresh cycle. On the other hand, Ashrafi reported that patients had higher implantation rates when they used FSH/LH rather than FSH alone (Ashrafi *et al*., 2011). More studies are yet required to look into the possible superiority of LH/FSH protocols at improving cleavage post-thaw rates.

In fresh cycles, endometrial thickness did not affect embryo survival or pregnancy outcomes of subsequent FET cycles. This is expected because the endometrium is being prepared for the implantation of a thawed embryo. One study, however, reported that patients with an endometrium thickness of 11.5 mm or less in fresh cycles maintained the same endometrial thickness in a subsequent FET. In these cases, additional endometrial preparation may be required (Jimenez *et al*., 2013).

Indication of freezing did not affect embryo or pregnancy outcome in our study. Eftekhar *et al*. (2014) also reported that it did not significantly affect clinical pregnancy rates. This is expected because regardless of the indication of freezing, more important factors such as embryo quality and endometrial preparation to implant a thawed embryo are at play.

Regarding fresh cycle outcomes, we found that patients who became pregnant after fresh cycles were more likely to become pregnant in FET cycles. Our results are in agreement with the findings of another study (Ashrafi *et al*., 2011) and might be explained by the fact that good quality embryos in fresh and frozen cycles lead to pregnancy in both cycles. On the contrary, one author concluded that patients who became pregnant in fresh cycles were less likely to become pregnant in FET cycles. The author explained that the best embryos would be chosen for fresh cycles, leaving behind less reproductively competent embryos for FET cycles (Doherty *et al*., 2014). Another author reported that fresh cycle outcome did not affect the pregnancy outcome of FET cycles (Bdolah *et al*., 2015). In addition, we found that fresh cycle outcomes did not affect embryo survival or progression after thawing, as also reported by El-Toukhy *et al*. (2003).

After studying different methods of fertilization in fresh cycles in relation to FET cycle outcomes, we found that IVF, ICSI, or both did not affect embryo or pregnancy outcomes, as previously documented in other studies (Ashrafi *et al*., 2011; Oehninger *et al*., 2000; Eftekhar *et al*., 2014). Other authors, however, reported that embryos derived from ICSI had lower implantation rates than embryos from IVF in FET cycles (10.9% v*s.* 25%, *p*<0.025), although their survival after thawing was similar. The authors attributed these findings to the effect of cryopreservation on embryos derived from ICSI on their implantation capability (Macas *et al*., 1998).

The number of embryos frozen did not affect survival after thawing. An author reported similar mean numbers of frozen embryos in groups with embryos with intact blastomeres and embryos that had lost up to 50% of the original number of blastomeres (6.7 *vs.* 6.2 embryos) (El-Toukhy *et al*., 2003). Furthermore, we found that fewer frozen embryos were correlated with higher pregnancy rates. This might be due to the fact that higher numbers of frozen embryos usually occur in cases of OHSS, where embryo quality is not necessarily good. Lower numbers of frozen embryos usually occur when good embryos are replaced in fresh cycles, and only excess embryos of good quality are frozen. Conversely, Bdolah *et al*. (2015) correlated clinical pregnancy with greater numbers of frozen embryos in fresh cycles (6.64±4.4 *vs.* 5.31±3.7, *p*=0.01).

We also found that the day in which embryos were frozen did not affect post-thaw embryo survival, although day 2 and 3 embryos had a stronger tendency to progression than day 4 embryos. Since day 5 embryos were not cultured or transferred on the same day of thawing, they were not included in the analysis. As in our study, in terms of embryo survival after thawing, other authors found no difference in survival when embryos were frozen on day 2 *vs.* 3 or day 5 *vs.* 6 (Sifer *et al*., 2006; El‐Toukhy *et al*., 2011). On the other hand, one study reported that embryos frozen on day 3 had significantly lower survival rates than embryos frozen on days 1 or 2 (Liu *et al*., 2012). Considering embryo progression, Liu reported that embryo survival decreased from days 1 to 3 (Liu *et al*., 2012). We also found that the day of freezing did not affect the pregnancy rate, as also noted for day-5 or day-6 embryos by El-Toukhy *et al*. (2011). On the other hand, it has been reported that cryopreserved day-3 embryos had better pregnancy outcomes than day-2 embryos, and blastocyst FET yielded higher pregnancy rates than day-3 embryos (*p*<0.001) (Sifer *et al*., 2006; Huang *et al*., 2014).

We found that the freeze-thaw interval did not have any effect on embryo survival or progression. Riggs *et al*. (2010) described that cryopreservation did not affect embryo survival after thawing, as human embryos seem to be stable with cryopreservation. In terms of pregnancy outcome, we found that embryos cryopreserved for more than a year yielded higher pregnancy rates. This is probably related to the fact that successful fresh cycles automatically delay the freezing cycle by at least a year, confirming that cryopreservation duration does not adversely affect embryos. One way to check this correlation involves adjusting the data and removing cases of pregnancy to identify significance. Unfortunately, this type of analysis requires a larger volume of cases.

Conversely, it was noted that a 25-35-day interval between freezing and FET was associated with better live birth rates compared to a 50-70-day interval. Several studies reported that duration of freezing and the time interval between freezing and FET did not affect pregnancy outcomes (Santos-Ribeiro *et al*., 2016; Ashrafi *et al*., 2011; Kassab *et al*., 2009; Riggs *et al*., 2010; Aflatoonian *et al*., 2013).

Unfortunately, our findings were limited by the small size of the sample included in the study. Our data might also have been influenced by the fact that all fresh cycles were offered to patients aged 35 year or younger. Larger studies are required to analyze other factors affecting FET cycle outcome and FET cycle parameters in particular.

## CONCLUSION

Our results showed that patients who became pregnant after fresh cycles were more likely to get pregnant in frozen cycles. In addition, patients who had more than a year between the fresh and the frozen cycle were more likely to get pregnant after the frozen cycle. Patients who were stimulated in the fresh cycle with combined FSH/LH and who had embryo cryopreservation on days 2 or 3 were more likely to get better embryo progression.
